# Organic versus Inorganic Arsenic in Herbal Kelp Supplements

**DOI:** 10.1289/ehp.10472

**Published:** 2007-12

**Authors:** Ari S. Lewis

**Affiliations:** Gradient Corporation, Cambridge, Massachusetts, E-mail: alewis@gradientcorp.com

Amster et al. (2007) reported findings from a case study involving a possible link between arsenic toxicity and the ingestion of a kelp-based supplement. The authors concluded that the arsenic-contaminated supplement was the likely cause of the neurologic, dermatologic, and gastrointestinal symptoms in their patient. Although the report has several methodologic shortcomings, the most serious flaw is the authors’ failure to recognize that the arsenic most commonly found in seaweed and seafood products is relatively nontoxic. This is in contrast to inorganic arsenic, which has well-documented acute and chronic toxicity. Amster et al. (2007) did not discuss the possibility that the arsenic measured in the kelp supplement was in the organic form, nor did they address the great variability in toxicity among arsenic compounds. These two oversights lead to the unsupported conclusion that the arsenic found in kelp is responsible for the unique set of medical conditions observed in their patient.

Amster et al. (2007) stated that “all chemical forms of arsenic eventually produce the same toxic syndrome.” In fact, the toxicologic properties of organic arsenic compounds are very different from those of inorganic arsenic. Inorganic arsenic is significantly more toxic than pentavalent arsenic compounds, arsenosugars, and arsenobetaine [Agency for Toxic Substances and Disease Registry (ATSDR) 2007b]. Arsenobetaine is a common constituent of seafood and is considered nontoxic. Interestingly, the major organic arsenic compounds in most seaweed are arsenosugars, which are still much less toxic than inorganic arsenic. For example, in an *in vitro* cytotoxicity assay, inorganic arsenic was 50 times more toxic than the trivalent arsenosugar and > 600 times more toxic than the pentavalent arsenosugar (Andrewes et al. 2004). In a recent article on speciated arsenic in seaweed, Rose et al. (2007) confirmed that inorganic arsenic levels in most varieties of seaweed are undetectable. Thus, the assumption that organic arsenic in the supplement could cause toxicity consistent with inorganic arsenic is scientifically unsupportable.

Although Amster et al. (2007) did not quantify an arsenic intake dose, they did use urinary arsenic levels to estimate exposure. They noted that normal levels of arsenic in urine are 50 μg/g creatinine (roughly equivalent to 50 μg As/L) and that their patient had an elevated urinary arsenic level of 85.5 μg/g creatinine. According to the Agency for Toxic Substance Registry (ATSDR 2000), normal urinary arsenic levels are 50 μg/L, but only “in the absence of recent consumption of seafood.” After seafood consumption, arsenic urinary levels can reach 1,000 μg/L (Vahter 1994). Thus, it is clear that 85.5 μg/g creatinine is not indicative of arsenic toxicity, particularly after known organic arsenic exposures. Many researchers have investigated the relationship between seafood consumption and urinary arsenic and have concluded that in order to make meaningful risk determinations through arsenic urine analysis, individuals should refrain from eating seafood (including seaweed) at least 4 days before testing (Foa et al. 1984; Kales et al. 2006).

Moreover, the symptoms most prominent in the patient described by Amster et al. (2007)—memory loss, alopecia, and fatigue—are not characteristic of arsenic toxicity (ATSDR 2007b; National Research Council 1999). The most sensitive non-cancer end point of arsenic exposure is the appearance of skin lesions (with very specific characteristics). Even these sensitive manifestations of chronic inorganic arsenic poisoning are not observed until lifetime exposures are hundreds of micrograms of arsenic per day (Abernathy et al. 2003).

There are several other limitations of the study by Amster et al. (2007). For example, the patient had manifestations of the conditions even before supplement use. Also, the authors did not discuss the possibility of iodine toxicity associated with the supplement ingestion. Certain comparisons the authors drew between the arsenic in the supplement and the regulatory limits are misleading. In particular, the reference to the Food and Drug Administration (FDA) food standard for arsenic of 2 ppm, which applies only to animals treated with veterinary drugs, is not relevant (ATSDR 2007a). FDA guidance recommends levels for seafood that are much higher. For example, the level of concern for total arsenic in crustaceans is 86 ppm, a concentration 10 times higher than the amount found in the kelp supplement (FDA 1993).

In conclusion, Amster et al. (2007) inappropriately relied on total arsenic data to link arsenic exposure to disease. They used their findings to comment on safety in the dietary supplement industry as a whole, implying that their results indicate that heavy metal contamination in supplements is a major health concern. Although contamination in food and dietary supplements is an issue that should be examined, their article did not inform this issue, and it obscures more significant food safety concerns that are of greater public health significance.
